# “They Would Have Stopped Births, if They Only Could have”: Short-and Long-Term Impacts of the COVID-19 Pandemic—a Case Study From Bologna, Italy

**DOI:** 10.3389/fsoc.2021.614271

**Published:** 2021-04-22

**Authors:** Brenda Benaglia, Daniela Canzini

**Affiliations:** ^1^ University of Bologna, Bologna, Italy; ^2^ Voci di Nascita Birth Community, Bologna, Italy

**Keywords:** birth, maternity care, COVID-19, covidian, reproductive rights, midwifery, public health, Italy

## Abstract

This article addresses the short-term impacts of the COVID-19 pandemic in Italy and hints at its potential long-term effects. Though many might want it to, birth does not stop during a pandemic. In emergency times, birth practices need to be adjusted to safeguard the health of birthing mothers, babies, birth providers, and the general population. In Bologna, Italy, one of the emergency measures employed by local hospitals in response to COVID-19 was to suspend women’s right to be accompanied by a person of their choice for the whole duration of labor and childbirth. In this work, we look at how this measure was disputed by the local activist birth community. Through the analysis of a social campaign empowered by Voci di Nascita—an association of parents, birth providers, and activists—we examine how social actors negotiated the balance between public health and reproductive rights in a time of crisis. We argue that this process unveils several structural issues that characterize maternity care at the local and national levels, including the (re)medicalization of birth, the discourse on risk and safety, the internal fragmentation of Italian midwifery, and the fragility of reproductive rights. The Covidian experience forced the reshaping of the birth carepath during the peak of the emergency. We suggest that it also offered an opportunity to rethink how birth is conceived, experienced, and accompanied in times of unprecedented global uncertainty—and beyond.

## Into the Field: COVID-19 in Italy

The first case of an Italian contracting COVID-19 was documented on February 21, 2020. “Patient 1” is a 38-year-old man from a small town near Milan whose wife was pregnant. In the turmoil of the breaking news, this detail was often repeated. Our thoughts—as a medical anthropologist and an activist mother engaged in the field of birth—ran to this mother-to-be, who was abruptly separated from her husband when he was confined to intensive care. We felt frightened and powerless, thinking about the health risk to her and their baby. Very little was then known about SARS-CoV-2, let alone about its effects during pregnancy. At that time, we could not imagine that separation, isolation, and loneliness would become trademarks of how we give birth—and die—during a pandemic.

This was also when the national hunt for the “culprit” (Moretti, 2020) began. Attempts were made to trace the chain of contagion, in the hope of exorcising the growing fear that it was too late to stop it. Before February 21, the only two confirmed cases of the novel coronavirus in Italy dated back to January 30 and were related to two Chinese tourists on holiday in Rome. Until then, the media portrayed the virus as something lethal but exotic, still distant enough to leave all of us living in privileged old Europe substantially safe. A lethal mix of ethnocentric shortsightedness, structural issues in some parts of the Italian National Health Service, and the imponderability of nature created the perfect environment for the virus to proliferate in the country.

“Patient 1” and his pregnant wife were the tip of an iceberg that would soon reveal its magnitude. At the end of February, a frightening scenario was emerging: thousands of people were exposed to the risk of being infected, including hundreds of women who were soon to become mothers. Hospitals—where 99.9% of births occur in Italy (Ministero della Salute, 2019)—were quickly identified as dangerous contagion hotbeds. Health services, including the entire birth carepath, required urgent reorganization. Protocols had to be rewritten and interpersonal relationships reshaped in light of the sudden need for social distancing. Drastic measures were to be implemented, as births could not be stopped.

## Ethnography in the Time of COVID-19: Engagement *vs*. Social Distancing

This study looks at maternity care in Bologna, Italy, during the Italian COVID-19 lockdown. To describe this lockdown, an early op-ed in the *New York Times* was provocatively titled: “Even Mass Is Canceled” (Parks, 2020). From March 9 to May 18, 2020, all citizens not involved in primary activities (such as health care, food production and distribution, vital logistics, law enforcement and security) had to follow one simple rule: stay at home.

From our own domestic quarantines, we observed the turmoil that was swirling around maternity care in Bologna through texts, calls, photos, and accounts coming from “outside.” These were the voices of soon-to-be parents and birth providers—hospital midwives in particular—confronting this new Covidian world and enduring its immediate effects. On April 17—a full month into the total lockdown—the association Voci di Nascita sent a formal letter to the local political and healthcare authorities in representation of parents, birth professionals, and birth activists^1^. The goal was to denounce the temporary suspension of women’s right to be accompanied by a person of choice during labor and childbirth in the city hospitals. Dozens of parents followed suit, enclosing a copy of the letter in their inquiries to the public relations departments of the hospitals where they were planning to give birth. The authorities responded, opening up a dialogue with the association and its members. Shortly after that, along with the gradual decrease in the emergency and the progressive systematization of the scientific evidence, the most restrictive measures were modified.

This article, like our engagements in the field of birth, is driven by our shared desire to contribute to fostering positive cultural change and social impact (Low and Merry, 2010). Such an aspiration proves more urgent than ever in times of social distancing and temporary restriction of reproductive rights: timely and informed critique is vital to the constant maintenance process that public health deserves in a democratic setting. This was also the primary driving factor for the social campaign empowered by Voci di Nascita and the reason why we decided to analyze that campaign and its repercussions on the community and on maternity care.

Anthropological work normally requires prolonged participation in the research field and direct engagement in relationships with interlocutors and research collaborators. Given social distancing measures, this was not possible for us. Therefore, we designed a short-term ethnographic research plan, which included two online questionnaires, in-depth conversations between we two authors, informal exchanges with local birth providers, participation in relevant webinars, and a review of the latest literature on the topic. Our study combines the analysis of data derived from such sources and unfolds on the basis of previous engagements in the field—both Daniela’s as an activist mother and Brenda’s as an anthropologist (Benaglia 2013, Benaglia, 2016, Benaglia, 2018, Benaglia, 2020).

## Online Questionnaires: Goals and Preliminary Results

For the purposes of this research, we designed and disseminated two different online questionnaires: one addressed to parents, the other to midwives. We narrowed our core sample to individuals directly related to the social campaign empowered by Voci di Nascita (people who had joined it, asked for information, expressed support, and/or shared spontaneous testimonies). All of our primary respondents had written at least once to the campaign’s official email address during the lockdown period (March-May). Additionally, parents had to have given birth during that time or immediately afterward. A secondary sample is composed of parents and midwives from outside of Bologna who had not been directly involved in the local campaign. Because of their efforts in reacting spontaneously to the survey, we decided to include their input in the broader context of our analysis.

Both questionnaires were open from July 4 to August 4, 2020 on the web-based platform Qualtrics. The invitation to participate in the questionnaire for parents was sent by email from the dedicated address of the association Voci di Nascita to 62 parents. Afterwards, we published a post on the Facebook page of Voci di Nascita, thus introducing the work to a broader audience. Information on the campaign and the link to the survey then circulated among secondary recipients outside Bologna.

We emailed the invitation to participate in the second questionnaire to 12 midwives, all of them active in the city of Bologna. The message included a request to forward the email to colleagues potentially interested in participating in the study. Another email was sent to the local College of Midwives and, a few days later, a post with the link to our survey appeared on their Facebook page. The announcement was reposted on Voci di Nascita’s Facebook page.

The goal of the questionnaire dedicated to parents was to collect stories of those who gave birth during the lockdown in Bologna and how the emergency restrictions reshaped their experiences before, during, and after the birth. We focused our queries on the presence/absence of the partner or support person during labor and childbirth, as this was the issue we were most interested in exploring. Most inquiries were directed to birthing mothers; however, a final question was dedicated to the partners (among our respondents these were all fathers, except for one, who was a second mother). We asked no personal details apart from the parents’ age, and whether it was the first birth. We set up the survey using the strictest anonymity settings, and no additional sensitive data was recorded.

The mothers’ questionnaire received 49 complete or partially complete responses (more than 60% filled in). These 49 accounts constitute the sample taken for analysis. Of these responses, 29 concern the Bologna area, and 20 are from other parts of Italy. The average age of the respondents (mothers) was 35 for the Bologna area and 34.6 for the whole national area. The average age of the partners was 36.3 and 36.7, respectively. The percentage of primiparas in the Bologna area was 22% and 30% for the whole national area. 27 of the Bolognese births took place in a hospital, 1 in a maternity home, and 1 at home. In the rest of the national territory, 15 births occurred in a hospital or clinic, three at home, and in 2 cases the place of birth was not declared. In Bologna, 2% of the respondents gave birth in a different place from the one planned and/or desired; this percentage rises to 6% in the rest of the country. Before the emergency, the desired labor companion(s) in Bologna was: the partner for 23 women, the partner and the parturient’s mother for 3, and the partner together with the midwife for 3. On the national scale, these numbers are 37, 6 and 6, respectively. In Bologna, 21% of women stated that they were left alone during labor and childbirth (similar to the national percentage, 22%).

The goal of the questionnaire dedicated to midwives was to gather their personal views on how obstetric practice and care changed in response to the pandemic and collect accounts of their direct experiences accompanying women and births in times of COVID-19. Particular focus in the midwives’ questionnaire was also on the temporary prohibition/limitation of partners in the birthing room; additionally, it addressed midwives’s needs during the crisis. Again no sensitive personal data was requested, aside from age and city. However, to better understand and contextualize the responses of our interlocutors, we asked midwives to describe their professional environment and experience, and to name the three words that they most associate with midwifery. Finally, we invited midwives to share their thoughts about the future of birth and maternity care.

The questionnaire for midwives received 18 full responses, constituting the sample taken for analysis. Of these, eight concern the Bologna area and the other 10 the rest of Italy. The average age of the respondents in the Bologna area is 31.6 years; the total national average age is 36.6. In Bologna, four responding midwives work at a hospital, 1 in a family clinic, and 3 as freelance professionals. These numbers at the national level are 9, 2, and 7, respectively. The three words most frequently associated with midwifery by respondents were “listening,” “empathy,” and “compassion.”

## The *Voci di Nascita* Birth Community and Its Social Campaign

Voci di Nascita means “voices of birth” and was founded on International Women’s Day of 2017. The birth of the association is related to the personal experience of becoming a mother and a doula of the founder and president, co-author Daniela Canzini. For her, the direct encounter with motherhood suddenly revealed a personal lack of “birth culture,” which, she felt, demanded an active stance at the individual and social levels. From its beginning, the association’s fundamental aim was to create and promote “culture” around birth and parenthood through various forms of social activism and services to birthing families. Midwives were progressively identified as the strategic actors of their local birth communities. Together with women, Italian midwives are directly engaged in the biosocial process of birth and largely operate within the biomedical environment. In the association’s view, midwives’ voices—largely unheard—called for a dialogue with parents, institutions, and within midwifery as well.

During the initial phase of the lockdown, dialogue appeared urgent yet almost impossible to achieve. This was the frantic time in which major emergency adjustments to the birth carepath were adopted, along with the broader reorganization of hospital spaces and services. Information on the new procedures for parents and staff appeared swiftly, increasing everyone’s anxiety and stress. Homemade signs popped up on the doors of maternity wards with vague communications such as “Due to the COVID-19 emergency, it is no longer possible to allow the accompaniment of women during the whole duration of labor.” In late March, a worried mother-to-be saw such an announcement during a prenatal visit at the hospital and sent a picture of it to Voci di Nascita to share her disorientation. Although other city hospitals had not employed the same restrictive measure (yet), the general feeling was that things could only worsen, and there was talk of the possibility of separating mother from child after the birth. Luckily, that did not happen. Eventually, on April 7, all three city hospitals adjusted to the strictest rule: the birth partner was not allowed during labor and was only to be admitted at the expulsion stage of birth. This left no choice to parents who, until then, could weigh their hospital options and decide to give birth where the partner or person of choice was still allowed to be with the mother and support her during labor as well.

The association decided it was necessary to demand that parents’ and birth professionals’ voices be taken into consideration, despite—and, in virtue of—the current emergency. A few midwives had already shared their concerns and expressed their feelings of being impotent, voiceless, and stuck in a violent defensive mechanism with no clear direction. On April 17, Daniela Canzini signed and sent out a letter to the authorities in charge of local health and hospital services, social politics (in the fields of welfare, infant rights, and birth), and to the president of the Emilia-Romagna Region.

The letter was successful in opening a dialogue and receiving formal feedback. Daniela was invited to be duly informed on the situation with the local health authorities. The most restrictive measures were corrected: starting from April 27, partners could be present from the beginning of active labor up until after the birth.

Of course, the campaign’s immediate impact should also be read in light of the progressive decrease in the COVID-19 emergency and the consolidation of the scientific evidence. However, the social campaign did mark a turning point and created an important precedent. Moreover, the letter hinted at several structural issues that characterize the local culture of maternity care and birth, which the experience of COVID-19 unveiled. In the following sections, we will discuss these, combining the responses given by parents and midwives to our questionnaires with selected abstracts from the letter itself.

## Separation and Prohibitions: Hospitals and COVID-19

Since the beginning of the pandemic, one of the risks associated with the reorganization of the birth carepath on the basis of the principle of social and physical distancing was the obstetric tendency to “revert back” to “deeply held belief systems” (Davis-Floyd, et al., 2020). This risk included the reinforcement of technocratic practices through the employment of restrictive measures. Davis-Floyd (2001, 2018) has identified “separation” as the underyling principle of the technocratic model of medicine (Davis-Floyd and St. John, 2001). Since modernization, rationalism, mechanicity, and determinism—all resting on the basic *principle of separation*—have significantly shaped Western scientific thought, and that of biomedicine in particular. One of the side effects of its development has been a progressive medicalization process, which affects multiple aspects of society through forms of biopolitical control of bodily experiences (Foucault, 1963; Canguilhem, 1966; Illich, 1976). Pregnancy and childbirth are no exception (Martin, 1987; Lock, 2004).

The “principle of separation” unfolds in the technocratic paradigm of birth, which is predominantly male-centered, sees the body as a machine, the birthing process as inherently mechanical and prone to dysfunction, the hospital as a factory, the baby as a product, and the environmental and relational aspects of childbirth as irrelevant (Davis-Floyd, 1987, Davis Floyd, 2001, Davis Floyd, 2003). Despite considerable progress toward less restrictive, more humanistic approaches and the revaluation of midwifery care, this model still shapes birth “management” in hospitals, and over-medicalization generally characterizes birth practices in the country, although with significant regional differences (Scavini and Molinari, 2015)^2^.

In pre-Covidian and Covidian times, hospital spaces, protocols, and hierachies do rest on the principle of separation, which is complementary to what we are calling the *principle of prohibition*. The biomedical choice to remove the birth partner from the birth scene shows that both principles were amplified in practice during the peak of the crisis.

Giuseppe Battagliarin, renowned obstetrician and president of the regional Birth Commission, recently suggested a connection between COVID-19, the hospital environment, and the “principle of prohibition.” During a public webinar^3^, he stated that “This virus has authorized more than any other the right to prohibit.” Battagliarin noted that COVID-19 turned ordinary things, such as walking around or shaking hands, into something forbidden—inconceivable in ordinary times. In his view, the power and authority of hospitals and doctors is structurally related to the power to set limitations and to prohibit. According to Battagliarin, the decision not to allow partners to participate in the whole birthing process partly stemmed from an “instinct” to prohibit, structured into the biomedical approach: a tangible instance of the aformentioned risk of regressing to former times when women were never allowed birth companions, their human rights were ignored, and birth practices were more controlling and less sensitive to women’s protagonism and psychosocial needs.

In his speech, Battagliarin also mentioned and praised a “letter from women”—probably referring to the campaign of Voci di Nascita—and synthesized the overall situation that health institutions were facing in the peak of the crisis. The ever-present inner risk inherent to the bodily experience of pregnancy and birth was confronted with the outer, diffused, and violent risk of contagion. Risk was everywhere: social and physical distancing became key, turning separation into the driving principle of all emergency measures, including those regarding birth companionship—despite long-established evidence on the importance of not leaving birthing mothers unsupported. The letter of Voci di Nascita repeatedly draws attention to the importance of continuity of supportive care throughout the birth process. It stresses that the continuous presence of a trusted person of choice is an undeniable right of all women, including during pandemics, and that removing this right is detrimental to the health of mother and baby, and to the bonding process:

There is no such thing as a moment which is more important than the other. Childbirth is a very delicate process that should be protected in the continuity of intimacy. [This process] is built over time, and requires minimum environmental changes, especially in the expulsive phase. The solution [to the current emergency] cannot be at the expense of the experience of those who are born, those who give birth, and those who are there to support the most delicate and powerful beginning of one’s social and relational life (Voci di Nascita, April 17, 2020).

The restrictive measures failed to consider the authoritative evidence available on birth companionship (Bohren et al., 2017) and its effects in biological and social terms—both in the short and long run. Moreover, during birth and the early stages of parenthood, the very concepts of separation and physical distancing represent “conceptual and biological nonsense” (Coscia et al., 2020), as childbirth, breastfeeding, and nurturing care necessitate close physical contact. Such early physical relations are the very first social relations as well, and both aspects have repercussions on babies’ neuro-cognitive development (WHO, 2018). Yet, in the frantic peak of the emergency, a semantic—albeit essential—*quid pro quo* occurred: the terms “visitor,” “relative,” “support person,” and “parent” were suddenly mixed, with the result that partners were cut off just where their relationship with their newborns normally begins (Coscia et al., 2020). Emergency procedures assumed that “the other parent” could be separated from the birthing mother and their newborn. These new restrictions and prohibitions implied that childbirth could be regarded as a single, specific moment that could be separated from the broader process of becoming parents, thus technocratically devaluing its relational, social, and political entanglements.

## Evidence, Risk, and Safety During the Emergency

During the early times of the emergency, it was difficult to navigate the scientific evidence on the new virus, which was “being produced, published, and disseminated at a rate never seen before” (Renfrew et al., 2020). For this reason, the Italian Istituto Superiore di Sanità^4^ issued systematic reviews weekly from the end of February. The final report, published on May 31, acknowledges that, initially, local health services had to react on the basis of their organizational availability and that until March, the scientific evidence was still poor and not always consistent (Giusti et al., 2020).

The Voci di Nascita letter acknowledged the medical staff’s efforts in responding to the unprecedented needs that appeared during the pandemic and expressed trust in the good conscience of decision-makers confronted with extraordinary responsibilities. The letter also raised questions as to how the evidence was interpreted and used to drive the implementation of emergency measures. For instance, the letter quotes the WHO infographics^5^ and abstracts from the guidelines for birth professionals issued by the Emilia-Romagna Regional Health Service, which suggested that one person could accompany the mother during labor and birth^6^. Why then limit the duration of the presence of the birth partner to the expulsion phase? On the basis of what evidence had similar hospitals in the same city proposed different rules?

Such inquiries recall the accounts of some midwife respondents to the questionnaire, who could not understand the rationale for restricting access to “husbands who had been with women up until 1 min before entering the hospital.” Many interpreted this security measure in terms of a poorly informed, reactionary rule devaluing parents’ rights and babies’ wellbeing—and also complicating midwives’ job during labor and birth. For instance, one midwife wrote:

Restricting access to the delivery room to fathers or an accompanying person has been detrimental to the mother’s rights, the newborn baby, the father. It has undoubtedly harmed the delicate process of birth at various levels. Increasing anxiety and fear in pregnant mothers, altering the dynamics and timing of labor and childbirth, exposing the mother to an excessive emotional and psychological burden postpartum, creating a fertile ground for emotional and psychological repercussions for the mother (Midwife #17).

Another commented that:

The exclusion of partners was an absurdity experienced with anguish by women who sometimes turned towards alternatives in the wake of fear instead of awareness—a measure with absolutely no scientific basis: an action against human rights, a violence against parents and babies (Midwife #7).

This midwife raises several crucial issues, including that birthing parents could end up changing their birthplace because of fear. A mother confirmed that she and her partner eventually changed hospitals because “she could not even think” of giving birth without her husband. Some parents in our study wrote that, during COVID-19, they felt out-of-hospital birth could be safer. However, no one planning a hospital birth actually shifted to homebirth. On the other hand, the women who gave birth at home (1 in Bologna and 3 nation-wide)—having previously decided to do so—said that their greatest fear was an emergency transfer to the hospital because they knew they might end up laboring alone.

22% of the birthing mothers in our survey declared that they were, in fact, left alone in the hospital during labor and birth. For example, one woman said that, although she had been assured that her partner could be there, he was only called into the room just after the birth. Partners usually remained outside the hospital premises, waiting to be called by the birthing mother herself or—more likely, given the circumstances—by the midwife on duty. A father said that this situation made him feel “powerless” and the typical scene is decribed by another man, who wrote:

I spent the whole night outside the hospital on the phone with my wife to make her feel my presence and give her courage, even though I was afraid. I left my wife at 9 p.m., and I saw her again at 3 a.m., after labor. I was able to experience the emotion of childbirth during the pushing phase. Despite the restrictions, I was lucky to be there and see my daughter being born (Partner #20).

Fear and luck are recurring expressions in both mothers’ and fathers’ accounts, and a midwife noted that:

Most women have an attitude of acceptance of the restrictions. Some of us received official complaints from inpatients when they realized that we broke the rules when necessary. We endured continuous disputes with our superiors, and we feared being reported by other colleagues. Nowadays, it is more us than women who have been asking for more openness and urging them to stand up for themselves. Fear has been the master, lately (Midwife #11).

This statement echoes the general feeling that most mothers expressed about their birthing experience: in the chaotic times of COVID-19, many women entered the hospital already “tired,” “stressed,” “fearful,” and “disillusioned.” Referring to the initial phase of the emergency, when protocols were not clear and could change from one day to the next, one mother said:

During the last few weeks of pregnancy, we feared that my partner could not be present at the birth. This made me really upset. Luckily, things changed shortly before the birth, and he was able to be there. Otherwise, I would have considered it as violence (Mother #25).

In their accounts, almost all mothers expressed disappointment with regards to the prohibition. Some of them specifically pointed at the impossibility of reaching the necessary intimacy with partners during childbirth because they could only be there for the very final moments. And yet—while expressing frustration, sadness, and loneliness—most birthing mothers accepted the restrictive measure for the sake of “safety” and because they saw no feasible alternative. Some also pointed to the fact that they were “lucky” this was not their first birth, suggesting a diffused perception that the quality of assistance and care is ultimately a matter of luck (Campisi, 2015), rather than a well-established right worth fighting for.

## Midwives at War

The “war” metaphor has been employed in mainstream media to describe the scenario generated by COVID-19, especially inside hospital wards. We chose to adopt this metaphor herein because it mirrors the words used by many midwives in our study when describing their experiences during the pandemic. Midwife respondents agreed that empathetic care is essential for birthing women. They were vocal in denouncing the risks connected to leaving mothers alone and depriving them of personalized midwifery care, which requires presence, empathy, and close contact. They also believe that such care is an essential safety factor, especially in critical times. Most midwives seemed to suggest that COVID-19 restrictions endangered a double right to quality midwifery care: for women to receive it, and for midwives to provide it, safely.

During the lockdown in Italy, the first “battle” for midwives began with instructions to abide by emergency measures “without being asked what they thought about them”—as the letter by Voci di Nascita points out. One midwife in particular felt that, by adhering to the new rules, she was (re)producing violence against women—suggesting the urgent need to further analyze the forms of obstetric violence (Quattrocchi, 2019) that birth practitioners simultaneously perform and suffer (Liese et al., 2021). Moreover, midwives in leadership and coordination roles highlighted their “frustration” at having to control their midwifery colleagues by forcing them into practices with which they disagreed. Hospital prohibitions hit midwives hard and, partially due to their lack of power and authority in the biomedical hierarchy (Davis-Floyd and Sargent, 1997), their response was weak.

Midwives’ second battle, shared with all other medical staff, was the initial lack or inappropriateness of personal protective equipment (PPE). This was particularly hard for freelance out-of-hospital midwives who had no direct access to PPE. Although facemasks were deemed necessary, midwives reported that these played a detrimental role in their relationships with women by disguising facial expressions; furthermore, recalling the experience of a birthing mother, a midwife commented that “birthing with a facemask was asphyxiating.”

The third and most structural battle midwives have had to endure over the past few months is their intra-professional conflicts. Respondents testifed to the fact that not all midwives disagreed with the most restrictive measures and that, because of their own fear and exhaustion, some actually thought that it was better to exclude partners from the birthing room. Generational issues should also be factored in, as younger hospital midwives tend to be more enthusiastic about their jobs. Older, more experienced midwives who had already fought for decades for mothers’ rights were often tired and disillusioned by the fact that women themselves sometimes only care about not feeling pain or having a “souvenir” photo of their birth. Unfortunately, none of these midwives filled out our survey and therefore we cannot further elaborate on their perspectives. However, a young midwife wrote:

The fact that there are no partners is seen by the majority as safer. For me, this emergency showed who loves their job, and who does it only for money. I am not saying that we should go out and die for midwifery, but neither that we have to carry out illogical and absurd procedures. For me it does not have to be like that: every mother is different, and we have to remember that we are not there to cure them but to accompany them. I think some of my colleagues are in burnout (Midwife #1).

Anxiety and burnout are very likely to increase during emergency situations. Adequate psychological support for hospital staff is structurally lacking in regular times, let alone during the pandemic. Yet women’s needs for emotional support and reassurance grow during times of crisis, and midwives should be adequately prepared to respond (O’Connell et al., 2020), even though “healthcare staff did not sign up to be heroes fighting on the frontline” (Renfrew et al., 2020).

The key to understanding this broader “cold war” is structural in nature and goes well beyond midwives’ experiences of COVID-19. It interrogates the very status of the midwifery profession in Italy, which has been defined a “semi-profession” due to a lack of collective identity and professional autonomy, and for its internal fragmentation (Spina, 2012). This “pre-existing condition” simply emerged more evidently during the pandemic. One midwife concluded her account by saying: “I have strong concerns about the future of my profession.”

More broadly, when questioned about the future of birth and maternity care in post-Covidian times, midwives raised a number of issues. Some were afraid that fear might normalize the strictest rules and that the medicalization of birth might increase at the expense of midwifery care. Others worried about the quality of virtual antenatal and postnatal care and feared that women will end up being even more isolated. Some hope for the development of social policies to support out-of-hospital birth for normal physiologic pregnancies. One midwife summarized the feeling of most respondents: “An unfortunate scenario has opened up: the little importance given to being born as a form of relationship.”

## Final Remarks: A “Message” to Consider

As elsewhere, as also demonstrated in other articles in this Special Issue, the experience of COVID-19 exposed pre-existing structural issues in Italian maternity care, especially within the hospital environment. Under the initial epidemiological pressure, emergency measures reshaped the birth carepath in ways that highlighted the delicate balance between safeguarding public health and guaranteeing reproductive rights (Yuill, 2020). In this sense, COVID-19 might be recalled as “a watershed moment for birth rights” (Drandić and Van Leeuwen, 2020).

Well past the first and hopefully last Covidian summer, a generalized feeling of uncertainty still characterizes the entire Italian scenario. There has not been a “second wave” of emergency and, therefore, no further lockdowns. The contagion at the national level seems relatively under control, particularly compared with other European countries (France and Spain, for instance). However, it is hard to make predictions for the upcoming weeks and months, and a serious threat remains. For this reason, at the Bolognese level, the “semi-restrictive” rules implemented in local hospitals after the campaign of Voci di Nascita still apply: women can be accompanied by a person of choice from the beginning of active labor only. According to informal testimonies we continue to receive, hospital staff seem to be more flexible than during emergency times, and their approaches—whether more or less medicalized—tend to resemble those in place before the pandemic. The situation could be rapidly shifting, and it is too early to confirm any stable changes in midwifery practice in the hospitals of Bologna, let alone at the broader national level.

In our study, we could not analyze what happened outside of the hospital during the emergency, in private homes, family clinics, and birthing centers. Parents and midwives who participated in our survey raised several themes that also deserve further attention, such as the need to design supportive out-of-hospital birth policies at the local and national levels (Quattrocchi, 2018); the role of partners during prenatal and postnatal care; the revaluation or deterioration of interpersonal relationships during quarantine; the virtualization of community services; the relationship between birth and death in times of crisis; and the risk of retroceding on progress in honoring women’s rights by reinforcing unbalanced domestic and affective workloads in family life; and other related issues (Coxon et al., 2020).

Our work suggests that the pandemic has been a touchstone, or pivotal moment, for local in-hospital birthcare. It shows how easy it is to go back to over-medicalized birth practices that had been considered outdated and not evidence-based; how fragile is the awareness of parents of their reproductive rights; how paralyzing is the internal fragmentation of the midwifery profession. On the other hand, the social campaign empowered by Voci di Nascita represented a strong example of activist strategy during emergency times. It pronounced the needs for clear communication of the new rules and protocols, appropriateness of evidence-based emergency measures, recognition of professional roles and responsibilities among birth providers, and the guarantee of parents’ rights. Respectfully, yet firmly, the letter demanded that the voices of all actors involved in the birth process be taken into consideration during times of crisis. It also created an opportunity to reclaim engaged parenthood.

The immediate achievement of the social campaign probably rests on the fact that it came from outside the hospital environment and that it was openly political. However, medium- and long-term effects of COVID-19 on maternity care practices and policies cannot be clearly foreseen and will be largely determined by the active and direct involvement of birth providers, health authorities, and parents in the aftermath of the emergency. In the words of one of the midwives in our study, it is a matter of whether the “message sent out by COVID-19 is understood, or not.” COVID-19 dramatically exposed structural issues that characterize the multilayered experience of giving birth and accompaning the births of babies and parents. Oddly enough, the virus forced an eye to the socio-cultural implications of the process of birth as rooted in—yet not limited to—the biological experience.

Postscript (December 2020). At the time of submission for review of this article (October 2020), a second wave of COVID-19 had not yet hit Italy and we anticipated that no clear predictions could be made and that uncertainty and fear were still dominant. Indeed, a second wave did hit Europe and Italy did not escape it. The semi-restrictive rules described in our work are still in place, and no further significant progress has been made in the longed-for engagement towards an alliance of the actors involved for the safeguarding of appropriate and well-rounded practices in maternity care.

## Data Availability

The datasets presented in this article are not readily available because, although anonymized, sources might be identified. Requests to access the datasets should be directed to brenda.benaglia@unibo.it.

## Footnotes

1 The full letter, in Italian, can be found on the association’s Facebook page (https://www.facebook.com/vocidinascita, accessed August 20, 2020). Abstracts of the letter cited in this article, as well as quotes from questionnaires, have been translated into English by the authors.

2 It must be aknowledged that, so far, at least deliveries among women affected by COVID-19 do not show an increase in medicalization procedures, such as unnecessary c-sections (Maraschini et al., 2020). While this is certainly an achievement, it must be highlighted that the vast majority of birthing women are not SARS-CoV-2 positive and are being exposed to an environment that, directly or indirectly, tends towards over-medicalization.

3 The online conference Becoming Parents Together in the Era of COVID-19 was organized within the framework of the activities of the PARENT project. The full recording is available at https://www.facebook.com/watch/?v=893690627805177 (accessed August 11, 2020). PARENT, an acronym for Promotion, Awareness-raising and Engagement of Men in Nurture Transformations, is a European project that aims at enhancing gender equality and reducing violence against women by promoting fathers’ nurturing care, starting from pregnancy itself (OECD, 2016; Luppi and Rosina, 2019).

4 The Istituto Superiore di Sanità (ISS) is the main center for technical-scientific research on public health in Italy. It serves the Italian National Health Service and the Ministry of Health. In this article, we use the website of the ISS and its publications as our primary reference for epidemiological data. Complete information on the impacts of COVID-19 in terms of cases, deaths, and recoveries in the national territory is produced and constantly updated by the ISS through an integrated surveillance system (https://www.epicentro.iss.it/en/coronavirus/sars-cov-2-dashboard, accessed August 10, 2020).

5 The WHO infographic states that “All women have the right to a safe and positive childbirth experience, whether or not they have a confirmed COVID-19 infection. Respect and dignity; a companion of choice, clear communication by maternity staff; pain relief strategies; mobility in labor where possible and birth position of choice” (https://www.who.int/reproductivehealth/publications/emergencies/Pregnancy-3-1200x1200.png?ua=1, accessed August 20, 2020).

6 The document was published on March 22, 2020 and is available online (https://www.saperidoc.it/flex/cm/pages/ServeAttachment.php/L/IT/D/1%252F3%252F2%252FD.1aa8e5fc6fd8fcdde5c6/P/BLOB%3AID%3D1402/E/pdf, accessed August 20, 2020).

## References

[B1] Benaglia B. (2016). “Doula e maternità tra spazio pubblico e privato. Considerazioni dal campo su attivismo, ricerca e cambiamento,,” in Going Public. Percorsi di antropologia pubblica in Italia. I. Severi. Editor Italia (Bologna, Italy: CIS—Dipartimento di Filosofia e Comunicazione; Università di Bologna), 65–88.

[B2] Benaglia B. (2013). La cicogna sono io. Una etnografia dell’accompagnamento alla vita nell’Ecuador contemporaneo. Intrecci. Quaderni di antropologia culturale. 1, 41–54.

[B3] Benaglia B. (2020). La cura invisibile: potenzialità e limiti della pratica della doula. Antropologia e teatro. 12, 59–83. 10.6092/issn.2039-2281/10885

[B4] Benaglia B. (2018). “‘Mothering the mother’: doulas and the affective space,” in Everyday world-making: toward an understanding of affect and mothering. Editors Lane J. Joensuu E. (Bradford, England: Demeter Press), 238–259.

[B5] Bohren M. Hofmeyr G. Sakala C. Fukuzawa R. Cuthbert A. (2017). Continuous support for women during childbirth. Cochrane Database Syst. Rev. 7, CD003766. 10.1002/14651858.CD003766.pub6 28681500 PMC6483123

[B6] Campisi R. (2015). Partorirai con dolore. Milano, Italy: Rizzoli.

[B7] Canguilhem G. (1966). Le normal et le pathologique. Paris, France: Presses Universitaires de France.

[B8] Coscia A. Cavicchioli P. Strola P. (2020). “Così lontano, così vicino”: nascere ai tempi del coronavirus Medico e Bambino pagine elettroniche XXIII, 95–98. Available at: https://www.medicoebambino.com/?id=CM2005_10.html.

[B9] Coxon K. Turienzo C. F. Kweekel L. Goodarzi B. Brigante L. Simon A. (2020). The impact of the coronavirus (COVID-19) pandemic on maternity care in Europe. Midwifery 88, 102779–102785. 10.1016/j.midw.2020.102779 32600862 PMC7286236

[B10] Davis-Floyd R. (2003). Birth as an American rite of passage. Berkeley, CA: University of California Press.

[B11] Davis-Floyd R. E. (1987). The technological model of birth. J. Am. Folklore. 100, 479–495. 10.2307/540907

[B12] Davis-Floyd R. Gutschow K. Schwartz D. A. (2020). Pregnancy, birth and the COVID-19 pandemic in the United States. Med. Anthropol. 39, 413. 10.1080/01459740.2020.1761804 32406755

[B13] Davis-Floyd R. Sargent C. F. (1997). Childbirth and authoritative knowledge: cross-cultural perspectives. Berkeley; London: University of California Press.

[B14] Davis-Floyd R. St. John G. (2001). From doctor to healer: the transformative journey. New Jersey, NJ: Rutgers University Press.

[B15] Davis-Floyd R. (2018). The technocratic, humanistic, and holistic paradigms of birth and health care. in Ways of knowing about birth: mothers, midwives, medicine, and birth activism by Robbie davis-floyd. Long Grove, IL: Waveland Press, 3–44.

[B16] Davis-Floyd R. (2001). The technocratic, humanistic, and holistic paradigms of childbirth. Int. J. Gynaecol. Obstet. 75 Suppl 1, S5. 10.1016/S0020-7292(01)00510-0 29645265

[B17] Ministero della Salute (2019). Certificato di assistenza al parto (CeDAP). Analisi dell’evento nascita–anno 2016. Available at: http://www.salute.gov.it/portale/documentazione/p6_2_2_1.jsp?lingua=italiano&id=2881.

[B18] Drandić D. Van Leeuwen F. (2020). COVID-19: a watershed moment for women’s rights in childbirth. Medical Anthropology Quarterly Rapid Response Blog Series. 1. Available at: http://medanthroquarterly.org/?p=536.

[B19] Foucault M. (1963). Naissance de la clinique. Paris, France: Presses Universitaires de France.

[B38] Giusti A. Zambri F. Marchetti F. Sampaolo L. Taruscio D. Salerno P. (2020). Indicazioni ad interim per gravidanza, parto, allattamento e cura dei piccolissimi di 0-2 anni in risposta all'emergenza COVID-19. Versione 31 maggio 2020. (Roma: Istituto Superiore di Sanitá. (Rapporto ISS COVID-19 n. 45/2020).

[B20] Illich I. (1976). Limits to medicine. Medical nemesis: the expropriation of health. London, England: Boyars. 10.1136/jech.57.12.928 PMC1732345 14652253

[B21] Liese K. Davis-Floyd R. Stewart K. Cheyney M. (2021). “Obstetric Iatrogenesis in the United States: The Spectrum of Disrespect, Violence, and Abuse.” Anthropology & Medicine, Special Issue on “Medicine’s Shadowside,” eds. Varley Emma Varma Saiba (in press). 10.1080/13648470.2021.1938510 34196238

[B22] Lock M. (2004). Medicalization and the naturalization of social control. Encyclopedia of Medical Anthropology., 116–125. 10.1007/0-387-29905-x_13

[B23] Low S. M. Merry S. E. (2010). Engaged anthropology: diversity and dilemmas. Curr. Anthropol. 51, S203–S226. 10.1086/653837

[B24] Luppi F. Rosina A. (2019). “From a single breadwinner model to two breadwinners to double earner/carer – do we need a new model?” in New visions for gender equality 2019. Editors Crowley N. Sansonetti S. Brussels, Belgium: European Commission, 20–24.

[B25] Maraschini A. Corsi E. Salvatore M. A. Donati S. (2020). Coronavirus and birth in Italy: results of a national population-based cohort study. London, England: British Medical Association 10.1006.11.2012865210.1101/2020.06.11.20128652 32959805

[B26] Martin E. (1987). The woman in the body: a cultural analysis of reproduction. Boston, MA: Beacon Press.

[B27] Moretti C. (2020). Il senso della colpa ai tempi del Covid-19. Milano, Italy: Nottetempo.

[B28] OECD (2016). Policy brief. Parent leave: where are the fathers? Available at: https://www.oecd.org/policy-briefs/parental-leave-where-are-the-fathers.pdf.

[B29] O’Connell M. Crowther S. Ravaldi C. Homer C. (2020). Midwives in a pandemic: a call for solidarity and compassion. Women Birth. 33, 205–206. 10.1016/j.wombi.2020.03.008 32241720 PMC7270587

[B30] Parks T. (2020). This is life under lockdown in Italy. The New York Times. Available at: https://www.nytimes.com/2020/03/09/opinion/italy-coronavirus.html.

[B31] Quattrocchi P. (2018). Oltre i luoghi comuni: partorire e nascere a domicilio e in casa maternità. Firenze, Italy: Editpress.

[B32] Quattrocchi P. (2019). Violenza ostetrica. Le potenzialità politico-formative di un concetto innovativo. EtnoAntropologia 7, 125–148. ISSN: 2284-0176.

[B33] Renfrew M. J. Cheyne H. Craig J. Duff E. Dykes F. Hunter B. (2020). Sustaining quality midwifery care in a pandemic and beyond. Midwifery 88, 102759–102767. 10.1016/j.midw.2020.102759 32485502 PMC7247475

[B34] Scavini M. Molinari C. (2015). in “Italy” in *Maternity services and policy in an international context: risk, citizenship and welfare regimes*. Editors Kennedy P. Kodate N. (New York, NY: Routledge), 179–204.

[B35] Spina E. (2012). An evaluation of the professional status of Italian midwives. Evid. Base Midwifery. 10, 88–93.

[B36] WHO (2018). Nurturing care for early child development. Geneva, Switzerland: World Health Organization.

[B37] Yuill C. (2020). Reproductive rights in the time of COVID-19. Somatosphere. Somatosphere, 1–7. Available at: http://somatosphere.net/2020/reproductive-rights-in-the-time-of-covid-19.html/.

